# Successfull management of a cervical oesophageal injury after an anterior cervical approach: a case report

**DOI:** 10.11604/pamj.2017.28.274.13870

**Published:** 2017-11-28

**Authors:** Omar Boulahroud, Abdelkrim Choho, Assou Ajja

**Affiliations:** 1 Departement of Neurosurgery, Military Hospital My Ismail, Meknes, Morocco; 2 Departement of Surgery, Military Hospital My Ismail, Meknes, Morocco

**Keywords:** Esophageal perforation, sternocleidomastoid muscle flap, cervical spine surgery

## Abstract

The anterior surgical approach for spinal repair, with or without the insertion of stabilizing hardware, is an established procedure in the management of anterior cervical spine (ACS) pathology. Esophageal injury during this approach is a rare complication that can be life threatening. No treatment protocol has yet been standardized. In addition to conservative measures, several surgical approaches have been presented, ranging from primary repair to reconstruction with local, regional, or distant flaps. The SCM muscle flap, used as reinforcement of a primary suture or as a patch to the lesion is in our opinion an effective treatment for persisting or recurring esophageal fistulae after anterior cervical spine surgery.

## Introduction

Esophageal injury during anterior cervical spine (ACS) approach is a rare complication that can be life threatening; generally it's the result of intraoperative manipulation, sometimes this can be secondary to hardware erosion and diagnosed months to years after surgery. Most authors agree that esophageal perforations are best treated by primary surgical repair, especially if recognized early but there is no standardized treatment protocol [1]. We believe that optimal management technique is the use of local, regionally pedicled and even distant flaps for achieving and maintaining perforation closure. In this case report we describe an esophageal perforation after an anterior cervical approach closed using sternocleidomastoid (SCM) pedicled muscle flap.

## Patient and observation

**Figure 1:**
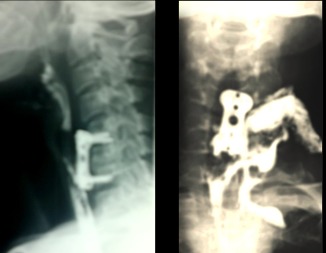
Esophagography with gastrograffin swallow showing contrast leakage through an esophageal fistula

**Figure 2:**
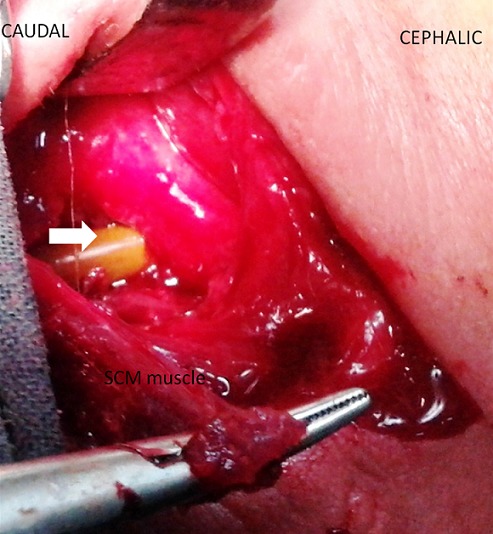
Intraoperative photograph showing an esophageal wall defect and a nasogastric tube (white arrow)

**Figure 3:**
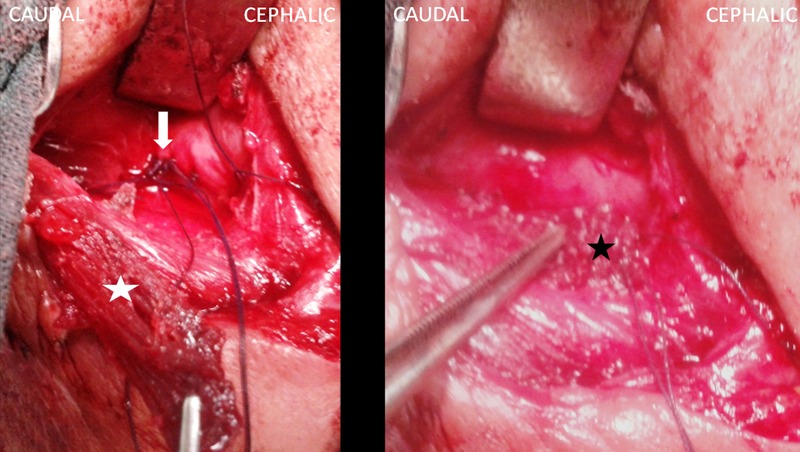
Intraoperative photographs: the esophageal fistula was closed using 3-0 Vicryl (wight arrow), then a pediculed SCM muscular flap (wight star) was elevated, medially rotated and interposed between the esophagus and the cervical column to protect the viscus repair (black star)

A 58-year-old women presented with symptomatic cervical neck and left arm pain. MRI of the cervical spine demonstrating prominent left sided C5-C6 disc herniation causing spinal cord and nerve root compression. She underwent by an antero-lateral left side cervical approach a C5-C6 dissectomy with interbody iliac bone graft and anterior cervical plate fixation. On the morning of the second postoperative day, she started to complain of dysphonia and dysphagia and presented a cervical swelling at the surgical incision site. Over the next 48 hours period, her clinical conditions worsened: she was found pyretic and moderately dyspnoic, the dysphagia turned into odynophagia, whereas the localized swelling diffused in the form of a mantle like subcutaneous emphysema of the neck. Gastrograffin swallow x-ray demonstrated signs of a pharyngo-esophageal rent as leakage of the contrast mean into the upper mediastinum (Figure 1). A consecutive attempted conservative approach, by administering wide-spectrum antibiotics, enteral feeding by nasogastric tube and a cervical subcutaneous drain led to only partial clinical amelioration, reduced the cervical swelling, but still the suction tube at the cervical surgical site draining corpuscolated material. Esophagoscopic finding shows the anterior cervical plate outside a thinner esophageal wall. As the fistula still persisted even after 45 days of conservative treatment and disapearance of inflamation, the patient was taken to the operating room. By a left lateral cervical approach, the fistula tract ahead of the neurovascular pedicle was followed deeply to the left lateral pharyngo-esophageal rent (Figure 2). The latter was found corresponding to the previously placed cervical column-blocking device. Once the remaining esophageal mucosa was isolated from the device, we decide to remove the cervical plate. The rent was primarily sutured with a reinforced 2-layer (mucosal and muscular) row of separated absorbable stitches. A superiorly based SCM muscular flap was then elevated, medially rotated and interposed between the esophagus and the cervical column to protect the viscus repair (Figure 3). The patient was kept on enteral feeding by the naso-gastric tube. Once the contrast swallow control fell to demonstrate any sign of esophageal rent, the patient started to take liquids by mouth and was discharged on the 11^th^ postoperative day. After one month a flexible endoscopic control confirmed the absence of esophageal perforation, while the patient is taking a normal oral diet.

## Discussion

Pharyngoesophageal injury after anterior spinal approach has been described since the development of the procedure. The most vulnerable pharyngo-esophageal site to lesions is the Killian triangle, which is formed by the inferior constrictor pharyngeus and the cricopharyngeus muscles. In this region, corresponding to C5/C6, the posterior esophageal mucosa is unprotected by muscularis and is separated from the retroesophageal space only by the buccopharyngeal fascia [1]. Another area of weakness is the lateral wall of the pyriform sinus at the level of the thyrohyoid membrane [2]. Review of the literature for esophageal injury reveals a range of 0% in Cloward's series [3] to 1.62% in Elerkay's corpectomy report [4]. But because of the delay in diagnosis-that can span from intraoperative recognition and repair to 10 years out-this complication is probably underreported. Presenting symptoms and signs, such as dysphagia, neck pain and swelling, hoarseness, cough, aspiration, dysphagia, odinophagia, subcutaneous emphysema and fever can be overlooked or suggest diagnoses of other more common local or systemic conditions. However, diagnostic or treatment delays significantly increase the risks of morbidity and mortality [5, 6]. The computed tomographic scan of the neck and chest is the most indispensable preoperative radiologic exam.

Common findings include air-fluid levels and subcutaneous emphysema. An esophagram is useful if additional information is required, mostly to pinpoint the site of the leak; however, barium studies can be negative in up to 25% of patients [6]. The value of radiologic tests is to provide an anatomic guide for proper debridement, while intraoperative endoscopy under general anesthesia is essential to delineate the site of the leak and whether or not a primary repair can be attempted. Immediate operative treatment of esophageal perforations is paramount. A non operative approach (percutaneous drainage, antibiotics and esophageal rest) has been reported in very small series, although operative drainage and repair remains the mainstay of therapy [7]. In our case the diagnosis was made two days after surgery with infection syndrome, that's why we first attempt a conservative treatement until resolution of the infection.We did not find in littérature any recommandation about the delay of secondary repair surgery. The best surgical procedures consist on use of muscular flap (sterno-cleido-mastoidian muscle, longus coli muscle,or pectoralis muscle) for reinforcement of the the suture. Primary closure with an SCM muscle flap is considered the standard surgical treatment for esophageal perforation, because the SCM is close to the cervical esophagus, is easy mobilized and is sufficient to cover most esophageal defects. Benazzo et al reported successful surgical repair using this flap [8], however some complication related to use of this flap as neck functional loss, myonecrosis or cosmetic alteration have been reported. The pectoralis myocutaneous flap may be used to close a cervical esophageal defect, but it presents the risk of loss of pectoralis functions. Rees et al used seven pectoralis flaps when closing chronic esophageal fistulas after tumor resections in cancer patients [8]. The longus colli muscle can also be used for closure of defects in the lateral pharyngeal wall or pyriform but it introduces the risk of injury to the cervical sympathetic trunk [9]. Moreover, Reid et al claimed that the omental free flap provides more successful outcomes than the pedicle flap for the closure of esophageal fistula after spinal surgery [10] this flap presents greater risks of microvascular thrombosis and loss of the flap.

## Conclusion

Esophageal perforation during cervical spine surgery is an uncommon occurrence. Care must be taken during exposure and instrumentation to minimize esophageal contact. When conservative treatment is not successfull aggressive surgical management is required. the SCM muscle flap, used as reinforcement of a primary suture or as a patch to the lesion is in our opinion an effective treatment for persisting or recurring esophageal fistulae after anterior cervical spine surgery.

## Competing interests

The author declare no competing interest.
